# Closure or Non-Closure of Peritoneum in Cesarean Section: Outcomes of Short-Term Complications

**DOI:** 10.5812/atr.8327

**Published:** 2013-02-01

**Authors:** Zohreh Tabasi, Mehrdad Mahdian, Masoumeh Abedzadeh-Kalahroudi

**Affiliations:** 1 Department of Obstetrics and Gynecology, Kashan University of Medical Sciences, Kashan, IR Iran; 2 Trauma Research Center, Kashan University of Medical Sciences, Kashan, IR Iran

**Keywords:** Cesarean Delivery, Pain, Surgical Diagnostic Techniques

## Abstract

**Background:**

Cesarean section (CS) is one of the most frequently performed surgical procedures worldwide. The complications following a CS include fever, wound infection, post-operative pain and bleeding which are not usually found in a normal vaginal delivery. Traditionally, suturing of peritoneal layers for CS patients has been done, but in some studies it has been shown that this procedure could be eliminated without affecting the rate of morbidity.

**Objectives:**

The objective of this study was to assess the short-term outcomes of two different cesarean delivery techniques.

**Patients and Methods:**

A total of 100 cases who underwent CS were randomly assigned equally to either closure of both the visceral and parietal peritoneum or no peritoneum closure. Duration of operation, pain scores, analgesic requirements, alterations in hemoglobin levels and febrile morbidity were assessed accordingly.

**Results:**

Pain scores, analgesic requirements assessed at 24 hours and operation duration were significantly lower in the non-closure group as compared to the closure group. Febrile conditions and changes in hemoglobin levels were similar in both groups.

**Conclusions:**

Non-closure of both visceral and the parietal peritoneum when performing a CS produces a significant reduction in pain, fewer analgesic requirements and a shorter operation duration without increasing the febrile morbidity and changes in hemoglobin levels as compared to the standard methods.

## 1. Background

Cesarean section (CS) can be considered as one of the most frequently performed surgical procedures worldwide, accounting for up to 70% of deliveries, depending on the facility being assessed and the country involved. In general, rates around the world are from about 5% to over 20% of all deliveries (1). In CS, surgical complications such as fever, wound infection, post-operative pain and bleeding occur more frequently than in normal vaginal delivery and these conditions may affect the postnatal care of newborn infants. Traditionally, suturing of peritoneal layers in CSs have been done, but in many randomized clinical trials, this stage could be easily eliminated since it does not increase the rate of morbidity (2-4). Reasons noted for closure of the peritoneum include restoring anatomy and re-approximating tissues, reducing infection by re-establishing an anatomical barrier, decreasing wound dehiscence, reducing hemorrhage and minimizing adhesions. Reasons cited for non–closure of the peritoneum include: reduction of operation duration, shortening of hospitalization admission, use of less analgesic, earlier return of bowel function, reduction of urinary bladder adhesion following next CS, and immediate post-operative recovery. It would also reduce the number of stitches which is the preferred option given that the body responds to stitches as if they were a foreign material (1-3, 5). Post-operative pain can cause unpleasant physiologic responses including retention of secretions in the respiratory system, ileus, increased usage of analgesics, increased post-operative stay in hospital and finally delayed breast feeding. Reduction of post-operative pain may improve mother’s comfort and eventually the outcome of newborn infant. Therefore, reduction of pain and use of fewer analgesics while still providing more comfort for patients is one of the important issues following CS. A series of studies evaluated the effects of leaving the peritoneum open and compared it with closing after CS. Some studies reported lower incidence of postoperative febrile morbidity, a shorter stay in hospital and an earlier return of bowel function following non–closure peritoneum compared to closure technique (3, 5). Other studies have not shown significant differences about wound infection, postoperative febrile morbidity and stay in hospital (4, 6-8).

## 2. Objectives

The present study was developed in order to study the controversial reports about the outcomes of closure versus non–closure of the parietal peritoneum following CS, and to compare postoperative morbidity of cited techniques.

## 3. Patients and Methods

This randomized double blind clinical trial was conducted in KashanShabih-Khani Hospital (Iran) in 2010 to compare the effect of closure with non-closure of both the visceral and parietal peritoneum on postoperative morbidity following CS. The study was approved by the Local Ethics Committee of Deputy of Research of Kashan University of Medical Sciences and written informed consent was obtained from each subject after discussing study objectives. One hundred women undergoing elective and emergency CS were randomly selected to receive either closure or non-closure of peritoneum, in accordance with a computer-generated random number list. Patients with former CS and/or abdominal surgery, diseases such as hypertension, diabetes mellitus and premature rupture of membrane and preoperative bleeding, were excluded. All patients received spinal anesthesia and were operated by the same surgeon. A transverse incision was employed in all the cases. In all cases, pain relief was obtained by rectal diclofenac (Diclofenac 100 suppository, Daru Pakhsh Pharmaceutical Mfg. Co -Tehran Iran) and intramuscular morphine. In the control group, both the visceral and parietal peritoneum were closed, whereas in the experimental group both peritoneal layers were left unsutured. The time of skin incision and surgery end time were recorded. At the end of surgery, 100 mg diclofenac per rectum and 5 mg morphine intramuscularly in addition to 3 doses of cefazoline were given to all patients. The end of surgery was taken as zero hours and pain was assessed thereafter at 6-, 12- and 24-hour intervals by visual analogue scale (0 mm = no pain, 100 mm = unbearable pain) by a nurse who was unaware of the surgical technique used. Mild (score < 30) and moderate pain (score 31 - 70) were managed with rectal diclofenac and severe pain (score > 70) was treated with intramuscular morphine. Both the rectal and injection’s analgesics were recorded for two days postoperatively. Hemoglobin and hematocrit levels of all patients were assessed prior and 12 hours following operation and the reduction of postoperative hematocrit up to 3 - 4% less than preoperative values was considered as bleeding. Patients were discharged on the third day following the operation. In cases with morbidities like fever, flatulence and spinal complications like headache and backache, the patient was not discharged and the reasons why were followed up and recorded. Analysis of data was performed with student's t-test and chi-square. P value less than 0.05 was considered significant.

## 4. Results

One hundred women undergoing elective and emergency CS under spinal anesthesia were randomly allocated in two equal groups, closure or non-closure. No significant differences were noted between the study groups with respect to age, parity, gestational age and reasons for CS (Table 1). Operative time was significantly shorter (6.89 minutes) in the non-closure group as compared with the closure group (Table 2). Febrile condition was recorded as 10% in the experimental group and 14% in the control group. This difference was not significant. One patient in the closure group developed endometritis and one patient in the non-closure group was diagnosed with mastitis which responded to antibiotics. There were no cases of wound infection in either of the two groups of the study. Moreover, there was no statistically significant difference between groups regarding surgical bleeding (38% in non-closure compared to 42% in closure group). None of the patients needed blood transfusions or a return to the operating theatre for any further surgery. Patients in the experimental group demonstrated lower pain scores (P = 0.0003) and used significantly less analgesics when compared with the control group (Table 2).

**Table 1:** Clinical Characteristics of the Patients Undergoing Cesarean Delivery by Either Closure or Non-Closure Technique

Clinical Characteristics	Closure (n = 50)	Non-Closure (n = 50)	P value
**Age,Mean ± SD**			NS a
Maternal age, y	25.2 ± 5.1	24.3 ± 5.2	
Gestational age,wk	38.83 ± 0.32	38.24 ± 0.52	
**Parity, No. (%)**			NS a
Primigravida	39 (78)	37 (74)	
Multipara	11 (22)	13 (26)	
**Main indication of cesarean delivery, No. (%)**			NS a
Fetal cause	17 (34)	18 (36)	
Maternal-Fetal cause	20 (40)	15 (30)	
Maternal cause	13 (26)	17 (34)	

^a^Abbreviations: NS, Not Significant

**Table 2:** Outcomes of Cesarean Delivery Using Either Closure or Non-Closure Technique

Factors	Closure, No. (%) (n = 50)	Non-Closure, No. (%) (n = 50)	P value
**Operative time, minute**			0.004
< 30	8 (16)	14 (28)	
30 - 40	23 (46)	31 (62)	
> 40	19 (38)	5 (10)	
**Pain score** a			0.0003
Mild	2 (4)	7 (14)	
Moderate	29 (58)	40 (80)	
Severe	19 (38)	3 (6)	
**Analgesic requirements**			0.0003
No need	24 (48)	41 (82)	
Rectal diclofenac b(No. of supp d)	21 (42)	7 (14)	
Morphine^c ^(No. of injections)	5 (10)	2 (4)	

^a^Pain score: Mild < 30, Moderate = 31 - 70, Severe > 70

^b^100 mg rectal

^c^5 mg intramuscular

^d^Abbreviation: supp, suppository

## 5. Discussion

This study showed that the non-closure of the peritoneum was associated with shorter duration of surgery, significantly lower pain scores and less analgesic use compared to traditional practice (closure of the peritoneum). In our study, the operative time was shorter (6.89 minutes) in the non-closure group than the closure group. A systemic review by Bamigboye and Hofmeyer revealed a reduction in operative time (7.33 minutes) in women who had both peritoneal surfaces unsutured in comparison with sutured peritoneum by analyzing a total of 6 studies with 947 participants (1). In addition to the cited study, a series of other studies also supported our findings about the reduction in operative time. There was a significant difference between two groups regarding pain scores and analgesic use in our investigation. Women in non-closure group had lower pain scores and received fewer analgesics. Diclofenac was used 3 times and morphine 2.5 times more in the control group compared to the experimental group. Rafique et al. in a randomized controlled study of 100 women (9) and Nagle et al. in a randomized trial of 549 women (5) reported less postoperative analgesia when the peritoneum was not sutured at CS. In the former study, pain was the primary outcome measure and investigators found no overall difference in pain scores between the two groups, although there was a trend of lower pain scores in non-closure group. In the latter study, analgesic use only was measured and authors found lower narcotic use in non-closure group. Both studies supported our findings. In our study, there was no significant difference between the two groups regarding postoperative febrile morbidity, wound infection and endometritis. Despite the lower incidence rate of fever and urinary infection in non-closure group in Nagele’s study (5), several studies did not show any significant difference regarding wound infection, endometritis, and fever between the closure and non-closure groups (1, 6-8, 10) which also supports our findings. In the present study, difference between pre- and post-operative hemoglobin level in both groups was not significant and neither set of cases required a blood transfusion. Malvasi et al. during the retrospective study of 2576 cases showed a significant increase of blood loss and transfusion in non-closure group (11). On the other hand, Nabhan reported significantly lower hemoglobin levels between preoperative and postoperative cases in the non-closure group versus the standard technique group while the blood transfusion rates in the two groups was comparable (12). A randomized controlled trial by Galaal and Krolikowski showed that estimation blood loss and mean drop in hemoglobin were not statistically significant between closure and non-closure groups (10). Many factors may contribute to the discrepancy between the results of our study and Nabhan’s and Galaal’s studies on one side and Malvasi’s study on the other side. Malvasi’s study is a retrospective study with a large sample size; however, our study and others are clinical trials with low sample sizes. Larger trials maybe required to compare the effects of bleeding in two different methods of surgery as one of the major complications of CS. The limitations of the present study should be recognized. For example, because of short duration of the study, long- term complications like adhesions were not considered and were outside of the scope of this study. A long-term evaluation of morbidity regarding adhesions is necessary to investigate the long-term complications of this approach. In conclusion, our study has confirmed that non-closure of both visceral and parietal peritoneum is associated with shorter operation duration, less pain, less demand for analgesia and is perhaps a preferred way to manage the CS patients because of these benefits.
